# Effect of blindfolding the lead resuscitator on frequency of closed-loop communication during veterinary cardio-pulmonary resuscitation training: a randomized, controlled pilot study

**DOI:** 10.3389/fvets.2024.1484506

**Published:** 2025-01-08

**Authors:** Olivia X. Walesby, Giacomo Stanzani, Lindsay Kellett-Gregory, Mayank Seth, Emily K. Thomas

**Affiliations:** ^1^The Royal (Dick) School of Veterinary Studies and the Roslin Institute, Easter Bush Campus, The University of Edinburgh, Midlothian, United Kingdom; ^2^Dick White Referrals, part of Linnaeus Veterinary Limited, Cambridgeshire, United Kingdom; ^3^Stansted Veterinary Services, Unit 7, Stansted Courtyard, Bishop's Stortford, United Kingdom

**Keywords:** closed-loop communication, CPR (veterinary), CPR training (veterinary), veterinary CPR scenarios, veterinary communication skills

## Abstract

**Objective:**

To evaluate the effect of blindfolding the lead resuscitator during veterinary cardiopulmonary resuscitation (CPR) simulation training sessions on frequency of completed closed-loop communication statements (CLC).

**Design:**

Ten groups of staff volunteers were recruited for a prospective, randomized, blinded, observational pilot study over a 6-month period. Additionally, two associated online questionnaires were completed by participants.

**Setting:**

Private veterinary referral hospital in the United Kingdom.

**Intervention:**

Forty volunteers were randomly allocated into ten groups of four. Each group was randomized as either control (CG) or blindfolded (BG) with the lead resuscitator always a veterinarian. The intervention involved the lead resuscitator wearing a blindfold during the third of four CPR simulation scenarios for the BG groups only.

**Measurements and main results:**

Video footage of Scenarios 2 (before) and 4 (after) the intervention was reviewed to quantify complete CLCs. Quantitative data were analyzed, and descriptive statistics calculated using GraphPadPrism (GraphPadPrism, Version9.3.1(350) forMacOSX, GraphPadSoftware, SanDiego, CA). Information from questionnaire responses was also analyzed. Statistical differences between the BGs and CGs were analyzed and there was no statistical difference in frequency of CLCs between the BGs and CGs during Scenario 2 (*p* = 0.76). In Scenario 4, however, following the intervention, there was a significant difference between BGs and CGs (*p* = 0.03), with a greater number of CLCs for the BGs compared with the CGs.

**Conclusion:**

Blindfolding the lead resuscitator in veterinary CPR training scenarios may be an effective method to increase the incidence of complete CLCs. Further studies would be required to investigate whether this finding is replicated and retained in the longer term.

## Introduction

**Figure 1:**
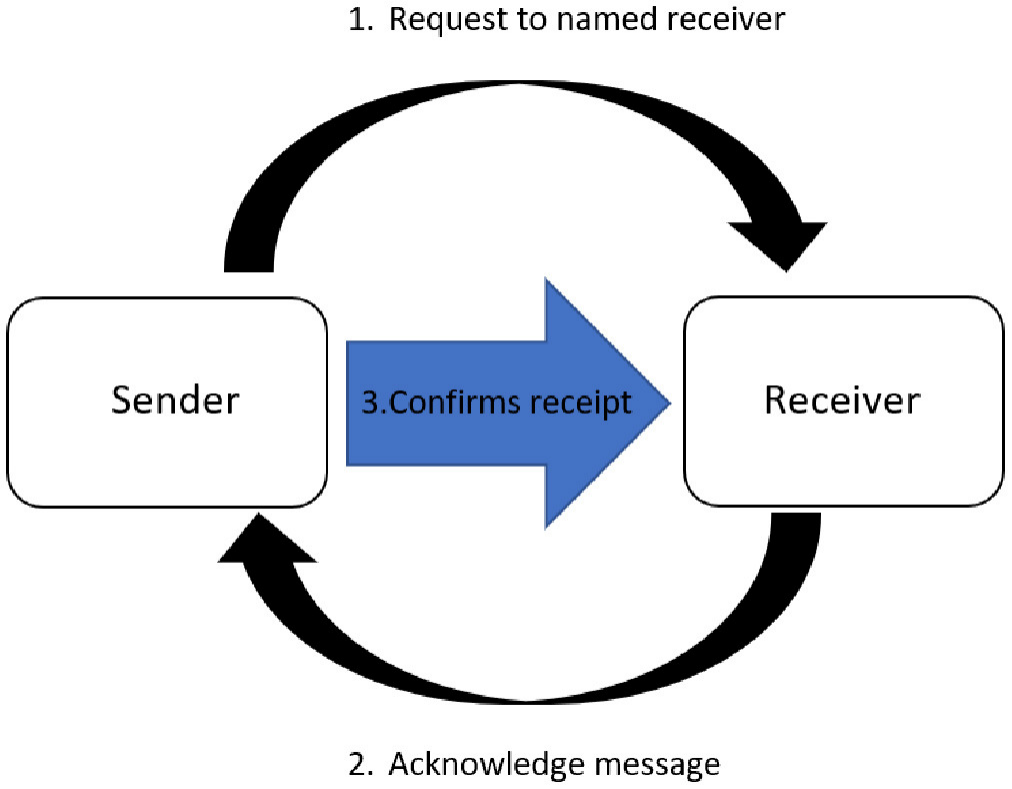
Closed-loop communication. (1) The sender requests an action to a named receiver; (2) the receiver audibly acknowledges the message; and (3) the sender audibly confirms receipt of the message.

CLC is a method of effective communication between individual team members in an emergency or while completing a critical task. CLC was used originally in military and aeronautical fields to implement effective communication, and later adopted by human and veterinary medicine particularly in resuscitations (1, 2). Clear communication during human resuscitation and trauma medicine along with other interventions are essential to avoid potentially fatal errors (3, 4). Improved communication such as CLC has been shown to improve human healthcare team performance in both simulation training and real-life emergencies (5–7). CLC comprises three components: (Part 1) the sender requesting an action to a named receiver; (Part 2) the receiver audibly acknowledging the message; and (Part 3) the sender audibly confirming receipt of the message (8, 9) (Figure 1). Successful CLC aids in reducing errors from miscommunication (10), not only by identifying a named receiver to whom the command is assigned, but also by having the receiver repeat back the request. CLC also facilitates a shared mental model for the team, as recommended by evidence-based human healthcare team performance frameworks (11). The Reassessment Campaign on Veterinary Resuscitation (RECOVER) CPR initiative suggests that the use of CLC during CPR may enhance team performance (1).

Despite these evidence-based recommendations for its use, both human and veterinary studies show a surprisingly low usage of CLC during real-life critical events and research observational settings (6, 12). One study from a veterinary teaching hospital reported that CLC was only used during six of twenty-two (27%) events (13). These data suggest that CLC may be a difficult skill to teach. Since current communication training techniques lack efficacy, investigation of novel techniques is warranted. Veterinary CPR simulation training sessions develop not only practical and technical skills but also communication skills such as CLC. The RECOVER CPR initiative conclude that team communication training may increase the efficacy of CPR teams (1).

In human medicine, recent studies (14, 15) reported CLCs increased when the lead resuscitator was blindfolded during training sessions. To the authors' knowledge, there are no studies examining CLC for veterinary teams during CPR training. The aim of the current study is to investigate the effect of blindfolding the lead resuscitator on the number of complete CLCs in veterinary CPR simulation sessions.

## Materials and methods

This prospective, randomized, blinded, observational pilot study was undertaken over a 6-month period at a private veterinary referral hospital in the United Kingdom. Ten groups of staff volunteers were recruited. Forty staff volunteers were recruited for the study via a hospital-wide email invitation. Inclusion criteria were current clinical role within the hospital (veterinarians, qualified veterinary nurses, student veterinary nurses, veterinary nursing assistants, and veterinary physiotherapists) and written consent submitted prior to commencement of the sessions. Exclusion criteria were CPR practical training received within the last 6 months and absence of a signed consent form. Recent CPR practical training would have given some study participants the potential advantage of practicing CLC more recently. Permanent emergency and critical care staff were excluded as they carry out CPR more regularly so inclusion of this group could also have led to bias. No participants had completed RECOVER Basic or Advanced Life Support Rescuer certification within the last 12 months but data was not collected on certification more than 12 months prior to the study.

Volunteers were randomly allocated to ten groups of four (labeled A-J) and each group was subsequently randomized as either control (CG) or blindfolded (BG) using an online random team generator and letter generator, respectively, by the secondary investigator (ET).^1^,^2^ The lead resuscitator was always a veterinarian and remained consistent throughout all four scenarios. Where groups contained more than one veterinarian, the lead resuscitator was randomly selected using an online random name-picker.^3^

Each volunteer was asked to complete a short pre- and post- study online questionnaire.^4^ The pre-study questionnaire was available 1 month prior to the session and the post-study questionnaire was available up to 2 months after the last CPR session had been completed. The questionnaires predominantly comprised of “yes/no” and factual questions to allow comparison of baseline characteristics between control and blindfolded groups. The pre-study survey gathered data on three areas: the participants' understanding of CLC, their usual role within the hospital and (for veterinarians) the number of years since graduation. The post-study survey asked participants about their understanding of CLC: whether they found the session helpful and a Likert scale to ascertain the likelihood of them using CLC again. A free text for feedback or comments was available. Participants were also able to communicate any concerns in confidence to the investigators. Partially completed surveys (started but not finished) were not available to the investigators. Questionnaires have been included in Supplementary material 1. Participants were asked to watch a 5-min online video outlining provision of basic life support according to the RECOVER guidelines (1) within the 24 h prior to the simulation sessions. The video did not discuss or refer to CLC.

CPR simulations were undertaken using a canine resuscitation manikin^5^ and simulation CPR box which included all equipment required for basic life support (BLS) and advanced life support (ALS) in accordance with the RECOVER guidelines (1). Water was substituted for drugs and drug bottles had needle-free valves to eliminate the use of sharps for health and safety reasons. Patient monitoring leads [electrocardiogram (ECG), end tidal carbon dioxide (ETCO_2_) and oxygen saturation probes (SpO_2_)] were also available for attachment, and were connected to a simulation monitoring screen displaying ECG and ETCO_2_. A copy of the RECOVER basic life support algorithm diagram and CPR drug dosage chart (1) was provided adjacent to the emergency drugs and reversal agents. Before the scenarios began, name badges were issued to each participant, and each team was given a 2-min period to familiarize themselves with the equipment and surroundings.

**Figure 2:**
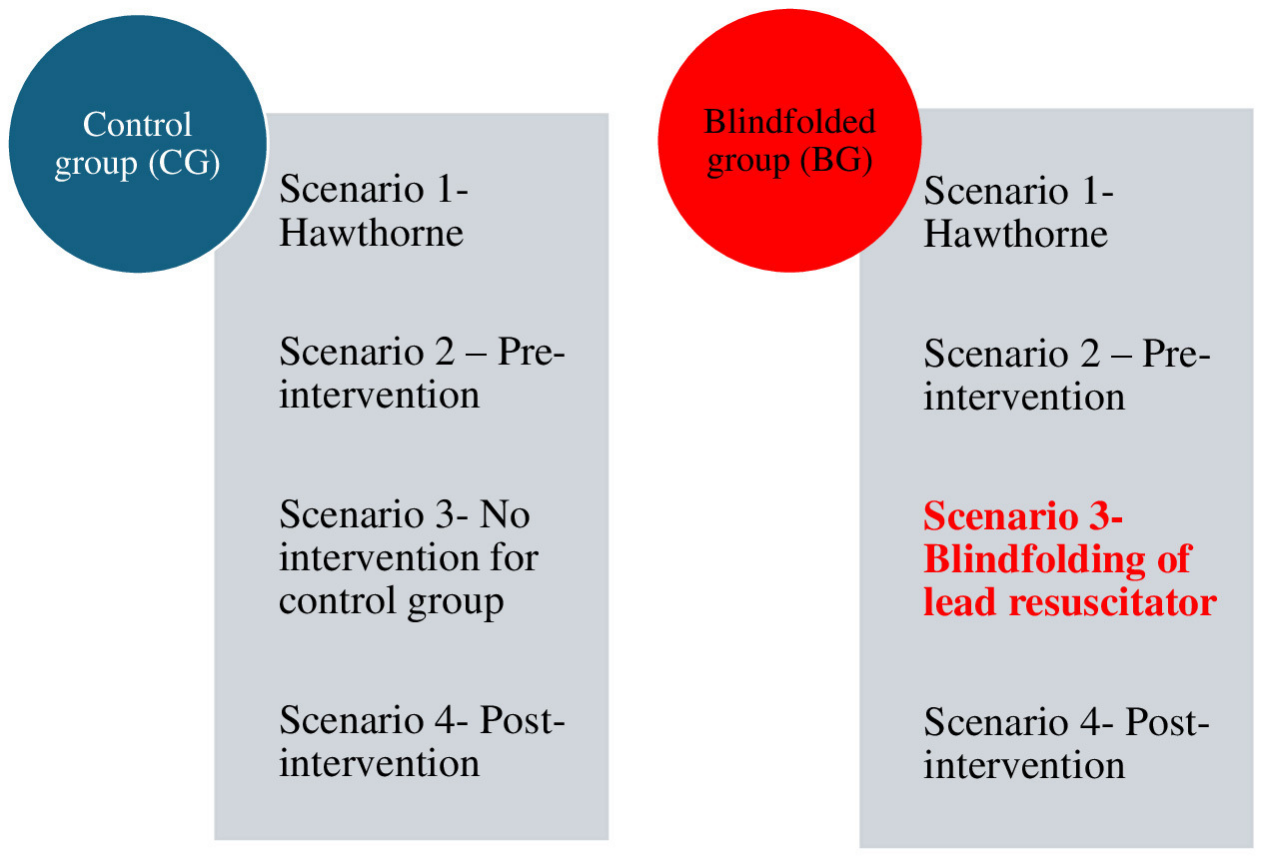
CPR scenarios.

Four standardized scenarios were undertaken for each group (Figure 2). The clinical description involved a canine patient that was presented with no history and no heartbeat and went into cardiopulmonary arrest immediately after administration of reversable sedation and a second that had the same presenting signs but no drug administration. The clinical descriptions were alternated so that Scenarios 1 and 3 had the same clinical description as Scenario 2 and 4 to allow comparison. The scenarios used are provided as Supplementary material 2. Once the participants connected the ECG leads, the simulation monitoring screen was set to asystole throughout all scenarios regardless of team performance. ETCO_2_ was set to 5 mmHg throughout. An initial 2-min simulation (Scenario 1) allowed subjects to acclimatize to being filmed. The remaining scenarios were each 5 min in duration. All scenarios were filmed using a 360-degree camera,^6^,^7^ but only Scenarios 2 and 4 were reviewed for data analysis. Scenarios 1, 2 and 4 were performed without blindfolding in either group. For Scenario 3, the lead resuscitators of BGs were blindfolded with a single-use eye mask. In contrast, lead resuscitators of CGs were not blindfolded. Scenarios were run by a secondary investigator who was not involved in data review (ET). Participants were invited to debrief on their team performance if they wished to do this: where performed, debriefing was not filmed or monitored.

For each scenario, a brief clinical description was read aloud to the participants after which a stopwatch and the video camera were started. Participants were told that for standardization purposes they could not ask the investigator any questions during the scenario unless they wished to withdraw from the study or address a concern for health and safety reasons. Participant(s) not wishing to take part in the session or deciding to withdraw at any point of the study were allowed to leave and their data deleted.

Filmed scenarios were reviewed by the principal researcher (OW) who was blinded to the intervention. The following data were recorded for each scenario: number of complete and incomplete CLCs, time to commencement of BLS according to the RECOVER algorithm [time to first chest compression (TTFC), time to first breath (TTFB)] and whether specific roles were assigned by the team leader.

Data were collated using a password-protected commercial database program,^8^ and anonymized by referring to group name before analysis. Complete CLCs were counted if the loop was completed without any other interruptions and if the receiver was named or if the role they were completing was named. Roles such as chest compressor and drug administrator were not assigned by the investigators. Other than the CPR leader role, participants could change roles within and between scenarios.

All sessions were completed according to government COVID-19 regulations at the time. This involved the wearing of surgical level face masks. Ethical approval was granted by the Royal College of Veterinary Surgeons ethical review committee (ERP 2021-25). The participants' consent included permission for presentations, publications, and discussions of the anonymized data.

### Statistical analysis

Due to the nature of the variables analyzed and the small population size, non-parametric statistics were used. Variables are described as median and range (minimum-maximum). Mann-Whitney *U*-test was used to compare variables and statistical significance was set at *p* < 0.05.

Numbers of CLCs were counted between the BGs and CGs after Scenario 2 and Scenario 4.

Mann-Whitney *U*-test was used to compare time in seconds for time to first compression (TTFC) and time to first breath (TTFB).

Survey data results are presented in a descriptive manner only as number (percentage) and, where appropriate median (range).

## Results

### Baseline data and group comparison

After recruitment and randomization there were ten groups in total: six blindfolded groups (BGs) and four control groups (CGs). Participants comprised of sixteen veterinarians, sixteen qualified veterinary nurses, four student veterinary nurses, three animal care assistants and one veterinary physiotherapist. No participants withdrew from the study.

The median number of years since graduation of the CPR leader was 7 years (range 3–10) for CG, and 6 years for BG (range 4–14). There was no significant difference between the groups (*p* = 0.614). Team leaders were recruited from interns (7), residents in surgery (1) and neurology (1) and an internal medicine specialist (1). English was the second language for 25% (10/40) participants and for 60% (6/10) of CPR leaders.

### Quantitative data

**Figure 3:**
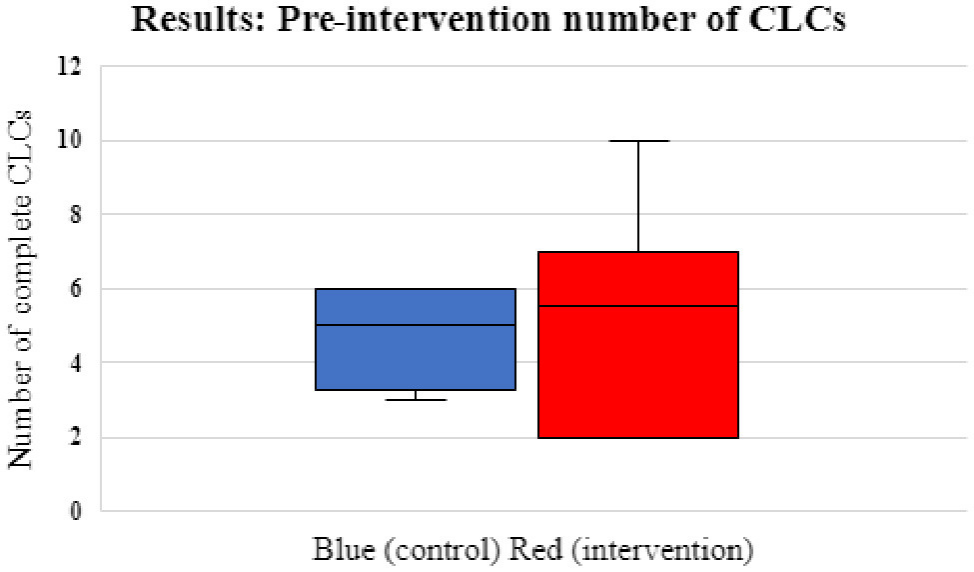
Box and whisker plots showing pre-intervention number of CLCs for control/CGs (blue) and intervention groups/BGs (red). The interquartile range (IQ 1 and 3) for the CG were 3.25 and 6. For the BG was 2 and 7. The median CLCs for the CGs was 5 (range 3–6) and the median for BGs was 5.5 (range 2–10) (*p* = 0.76).

**Figure 4:**
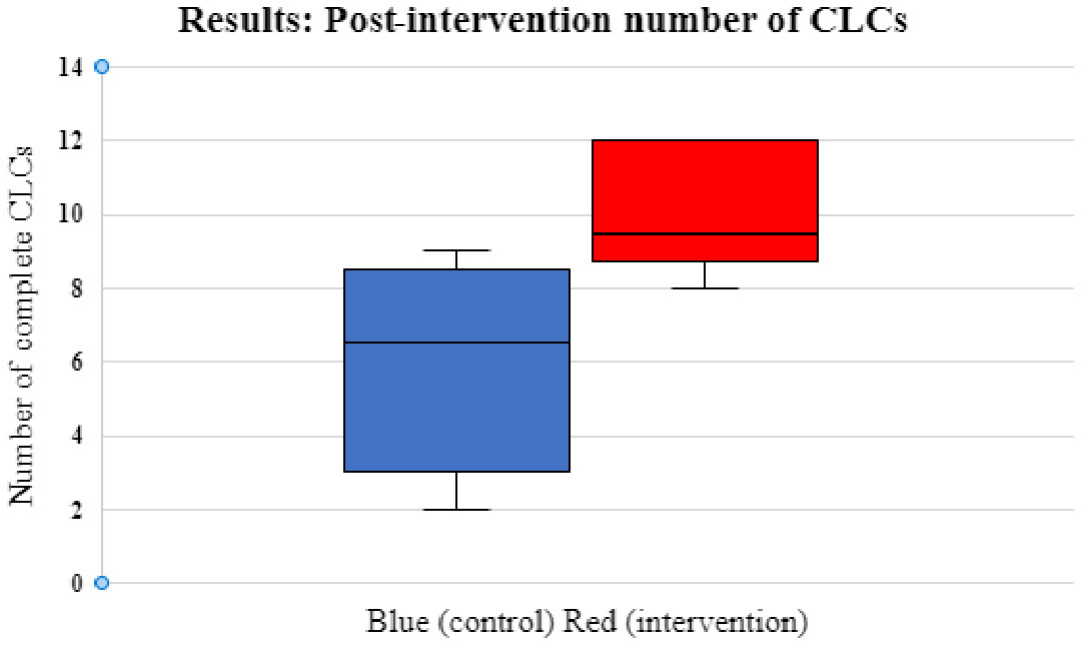
Box and whisker plots showing post-intervention number of CLCs for control/CGs (blue) and intervention groups/BGs (red). The interquartile range (IQ 1 and 3) for the CG were 3 and 8.5 and for the BG were 8.75 and 12. The median CLCs for the CGs was 6.5 (range 2–9) and the median for BGs was 9.5 (range 8–12) (*p* = 0.03).

Prior to the intervention (Scenario 2), the median number of CLCs in CGs was 5 (range 3–6) and in BGs 5.5 (range 2–10) with no significant differences between the two groups (*p* = 0.76). Following the intervention (Scenario 4), the median number of CLCs in CGs was 6.5 (range 2–9) and in BGs 9.5 (range 8–12). The difference was statistically significant with BGs completing more CLCs (*p* = 0.03) after blindfolding. Data on CLCs for the BG and CG pre- and post-intervention are shown in Figures 3, 4. CLC was most commonly used when confirming drug dosages or nominating the next person to take on the role of chest compressions.

Numbers of incomplete CLCs (sender and receiver with no confirmation from the sender) and complete CLCs are summarized in Tables 1A, B. The median incomplete CLCs for the CGs in the pre-intervention scenario was 6.5 (range 5–8). For the BG group the median incomplete CLCs for Scenario 2 was 9 (range 7–11). In Scenario 4, the median incomplete CLCs for the CG was 6 (range 3–9). In the post-intervention scenario for the BGs the median incomplete CLCs was 8 (range 5–11).

**Table 1A:** Numbers of complete and incomplete CLCs in the control group.

	**Scenario 2**		**Scenario 4**	
**Group**	**Incomplete**	**Complete**	**Incomplete**	**Complete**
B	6	3	7	2
C	7	4	5	6
D	5	6	3	9
F	8	6	9	7

**Table 1B:** Numbers of complete and incomplete CLCs in the blindfolded group.

	**Scenario 2**		**Scenario 4**	
**Group**	**Incomplete**	**Complete**	**Incomplete**	**Complete**
A	11	2	5	8
E	7	2	8	10
G	7	5	8	12
H	10	6	8	9
I	8	10	11	12
J	10	6	9	9

Data for TTFC and TTFB are summarized in Table 2. There was no significant difference between the groups for TTFC or TTFB.

**Table 2:** Mean time to first compression (TTFC) and time to first breath (TTFB) for control vs. blindfolded groups: scenarios 2 and 4.

**Scenario**	**Variable**	**Control group mean (seconds)**	**Blindfolded group mean (seconds)**	***P*-value**
2	TTFC	8.25	4.7	0.543
4	TTFC	3.74	5.8	9.305
2	TTFB	58.8	54.3	0.762
4	TTFB	46	33.5	0.614

Role assignment by the lead resuscitator in Scenarios 2 and 4 was analyzed. In the CGs, 2/4 groups assigned roles in Scenario 2 and 3/4 groups assigned roles in Scenario 4. In the BGs 2/6 groups assigned roles in Scenario 2 with 4/6 groups assigning in Scenario 4.

### Survey data

The majority of participants (33/40, 82.5 %) responded to the pre-study survey (Supplementary material 1). Participant responses were reviewed by the primary investigator (OW). When asked whether the participant understood what CLC was, 30/33 (90.9%) responded “yes” and 3/33 (9.1%) answered “no”. However, when asked to define in one sentence (free text) what the participant thought CLC meant, only 4/33 (12.1%) correctly identified the three stages of communication. Participants most commonly defined CLC as clear communication but without mentioning repetition, cross-checking or the three parts of CLC, and 5/33 (15.2%) participants responded that CLC should be closed questions with a yes/no answer.

Post-study surveys were completed by 28/40 participants (70%). Responses showed that 27/28 participants (96.4%) agreed the simulations were a useful exercise regardless of blindfolding status.

Participants were asked the question “How likely on a scale of 1–10 are you to use closed-loop communication in the future?” with a Likert scale rating (1 being very unlikely and 10 being highly likely). The median rating for this question was 10 (range 5–10). When invited to give feedback, the overall response was entirely positive. The response rate was above half for pre- and post-scenario (82.5 and 70%, respectively). No negative comments were received.

## Discussion

This pilot study investigated whether blindfolding groups of participants during veterinary CPR simulation scenarios influenced CLC usage. Blindfolding was chosen for investigation in this study due to its low-cost and simplicity allowing for easy implementation into training programs, including in resource-poor settings. There is also some limited medical literature suggesting positive results when trialed in pediatric resuscitation training (14). When the lead resuscitator is blindfolded, key visual cues are removed, forcing the team leader to rely on spoken cues instead. CLC usage was chosen for analysis as an easily quantifiable measure of communication, but it is hoped that the increased use of spoken cues may also benefit team dynamics in general by enhancing a shared mental model which is key to effective team performance (11). Blindfolding is clearly impractical for use in real-life CPR situations; therefore, it would only be a useful training tool if the effect persists after blindfolding. The principal finding in this study was a statistically significant increase in CLCs in the simulation scenario performed immediately after blindfolding, which seems to suggest a persistent effect at least in the short term. Due to COVID restrictions and the size of the participation pool available, this study proceeded as a veterinary pilot study for proof of concept.

Clear, effective communication in busy, dynamic settings such as hospitals is important. Communication issues have been associated with significant medical errors, morbidity and mortality (4, 9, 16). Clear communication has been highlighted as a key theme to improve resuscitation training in human medicine (16). CLC is an evidence-based communication technique that has been shown to increase efficacy of communication and safety across numerous industries including aviation, the military and, more recently, human healthcare (17, 18). Use of clear communication within a resuscitation team is thought to increase team efficacy as described above, and it forms part of the recommended communication strategy in the human and veterinary CPR guidelines (1, 2). Better communication including CLC has been associated with higher performing human hospitals for in-hospital cardiac arrest (16). However, implementing effective teaching of this skill can be challenging, and studies suggest it is under-utilized in real-life resuscitation scenarios (2, 14, 15).

CLC is typically defined as consisting of three parts (Figure 1) (6, 9). However, there are variations within the literature with some sources describing only two parts (sender requests, receiver acknowledges) (1). The pre-study survey highlighted that although most study participants were familiar with the term itself, there was considerable lack of understanding of its exact meaning. This should be taken into consideration when designing standard operating procedures and training programs within individual hospitals so that a preferred definition can be chosen and disseminated during training. Participants in this study received no training on the use of CLC.

Interestingly, it was noted by the lead investigator that CLC was most frequently used in relation to drug administration, or when a team member was being assigned as the next to commence chest compressions. There was no clear indication for this finding, although increased CLC use was similarly noted in relation to medication orders in an observational human study (12). One explanation for the increased using of CLC during these circumstances could be that when non-veterinarians are interpreting instructions from veterinarians they are more likely to use CLC to confirm understanding (i.e., already there is a norm for orders given and orders followed). CLC was less frequently used when assigning initial roles of compressors and ventilators, perhaps because team members had already automatically assumed some of these roles. However, in this case CLC would be expected to remain unchanged or even decrease with repetition of the scenario. It also appears that the number of roles assigned in Scenario 4 compared with Scenario 2 increased for both CGs and BGs. The cause of the increase of role assignment seen in the CGs is unknown, but the greater increase in BGs suggests that the blindfolding intervention may encourage the lead resuscitator to assign roles. The task of assigning roles leads to clear communication and the benefits previously highlighted as well as improved team performance (19).

As a secondary objective this study evaluated whether blindfolding the lead resuscitator influenced time to onset of BLS measured as TTFC and TTFB. There was no statistical difference between the groups. Both parameters (TTFC and TTFB) were chosen because they are easy to quantify and provide a crude indicator of adherence to the BLS algorithm. However, complete assessment would require measurement of many other factors such as frequency of chest compressions and breaths given (using visual feedback) and depth of chest compressions (e.g., using thoracic impedance devices) (20, 21). It is also important to recognize that, as with any simulation study, results cannot necessarily be extrapolated to a clinical CPR situation. Simulation scenarios and filming may both cause participants to consciously or unconsciously behave differently from the way they would behave in a real-life CPR situation. A high-fidelity manikin and resuscitation equipment were provided for the simulation scenarios, but fidelity was limited by having to conduct the study away from the clinic floor. Multiple scenarios were filmed with the aim to diminish the Hawthorne effect whereby participants may change their actions when they are aware of being watched (22). Nonetheless, it is difficult to completely remove the effect although the inclusion of control groups should have mitigated any residual effect. Team performance improves when CLC and clear communication improves (6, 10) and while this improvement does not necessarily translate to an improved clinical outcome it is logical to conclude that it would. Importantly, the study scenarios also did not fully address the complexity of leadership skills during CPR, which extend well beyond CLC. Leadership courses can improve team performances and enhance patient care (23).

The groups for this study were randomly selected from volunteer participants, thus participants were not in their normal team groups, which may have impacted communication including CLC usage. However, this reflects real-life variation which would be applicable across many large veterinary hospitals and emphasizes the importance of good communication training including CLC within the crash team. All volunteers wore name badges to avoid any barriers to using the recipient's name when sending the initial call-out.

This study had a higher survey response rate (82.5% for the pre-survey and 70% for the post simulation survey) than that found in some human studies although this can be variable depending on survey type and topic (24, 25). It is worth noting that not all participants filled in both questionnaires as some participants failed to fill in the follow-up survey. It is also possible that participants misunderstood individual questions. This could have affected the results, responses and our interpretation although, given the high response rates, any effect would likely be small. The questionnaire also did not ascertain the perceived readiness of veterinarians to act as leaders during veterinary CPR. Future studies would be needed to determine if further and more regular training increased the skills and ability of CPR participants and CLCs. Years post-graduation of the leader were not significant between the two groups. More recently graduated veterinarians may have had an advantage of more recent training, but older veterinarians may have had more experience acting in a leadership capacity as well as experiencing more CPR. Ascertaining more information from participants about previous leadership of CPR or leadership training may be useful in future studies.

The study investigators were initially apprehensive that participants might not be willing to be blindfolded. Blindfolding as a training resource may be underused, due to participant reluctance (6). However, not only did all participants willingly take part and consent to the sessions including blindfolding, where applicable, but the post- scenario comments were universally favorable reflecting that participants found the sessions useful. No participants asked to leave filming or the study. This further shows the acceptance of blindfolded simulation scenarios as a study format by a variety of veterinary staff and willing participation. There were no health and safety issues to address during the study. Due to incomplete survey responses, it is not possible to assess whether those who were in the BGs found the study more useful to increase frequency of CLCs than those in the CGs.

There are several limitations with this study. A key limitation was that the number of participants in each group and in the study overall were constrained by COVID restrictions at the time in the United Kingdom, and this study is therefore presented as a pilot. However, we felt that the number of people in a group, four, was sufficiently large to carry out appropriate CPR and to require CLC within the team. A minimum of one person can carry out basic life support in veterinary medicine (26). The optimal number in veterinary medicine for advanced life support appears inconclusive but in human literature a team of five people for out-of-hospital CPR (27) and seven people for pediatric trauma in-hospital resuscitation (28) are suggested to be optimal. In human hospitals there are often dedicated resuscitation teams that are multi-disciplinary (16), however, this is unlikely to be achievable easily in veterinary medicine. It is prudent to note that more people in a CPR scenario can also lead to more confusion and inefficiency although some human studies looking at task-based efficiency in resuscitations did not find this (29). Another limitation is that the total number of BGs were more than the CGs, four total groups of CGs and six total groups of BGs, due to rescheduling a group. Before each session, there was randomization of the group, as per the study protocol, and this led to unequal total number of groups. Whilst no participants had completed recent (< 12 months) RECOVER BLS or ALS Rescuer certification, knowledge of prior RECOVER certification or course enrolment was out with the scope of this study but could have led to bias.

Despite COVID restrictions, combined with the clinical requirements and normal staff turnover of a large, private referral hospital we were able to assess the main research question. We were unable to ascertain whether the increase in CLCs in the blindfolded groups was retained in the longer term e.g., by replicating simulations at different time points after the original study. The longer the post-blindfolding effect persists, the more useful blindfolding would be to include in simulation training. Identifying the duration of the effect in order to guide frequency of blindfolding teaching interventions would be an interesting area for future study. Multicenter studies may help recruit sufficient participants for any future studies. However, in busy referral centers with changing staffing levels, there would be challenges to obtaining a study population that was available at certain time points.

A range of important considerations arose both prior to and during the study, which may help to inform future study design. In particular, at our hospital there is a wide range of nationalities with 25% of participants and 60% of team leaders being non-native English speakers. Instructions were sometimes clarified by the responder or other team members if they were not clear, for example if there was need for type of drug or drug dose clarification required.

As there was only one video reviewer there is a possibility of bias in assessing CLCs although the principal investigator was blinded to group intervention. In human studies two video reviewers were used (14).

Despite these limitations, this study technique appears to be a non-expensive and engaging modality to increase the use of CLCs, which was very well-received by participants. The authors would encourage other training centers to consider similar projects to improve CLC during veterinary CPR training. It is still unknown how well the results of blindfolded simulation training transfers to real-life scenarios and the duration of this effect.

## Conclusions

In conclusion, our pilot study suggests that blindfolding the lead resuscitator in simulation CPR training may increase the frequency of CLC, and that simulations including blindfolding were not only economical and easy to facilitate but also positively perceived by participants. Blindfolding the lead resuscitator during simulation training could be a simple way to highlight the importance of effective team communication and could be considered during veterinary CPR simulation training. The principal finding was a statistically significant short-term increase in the number of CLCs in the BGs after the blindfold intervention compared with the CGs. Thus, further investigation into the longer-term effects of this novel and interactive training measure is warranted.

## Data Availability

The original contributions presented in the study are included in the article/Supplementary material, further inquiries can be directed to the corresponding author.

## Footnotes

^1^https://commentpicker.com/random-letter-generator.php (Nieuwveen, The Netherlands).

^2^https://commentpicker.com/team-generator.php (Nieuwveen, The Netherlands).

^3^https://commentpicker.com/random-name-picker.php (Nieuwveen, The Netherlands).

^4^SurveyMonkey Inc., San Mateo, California, USA.

^5^JerryK-9CPRmanikin, RescueCritters, SimiValley, CA.

^6^Ricoh Theta S ™, Ricoh Company Limited, Tokyo, Japan.

^7^GoPro Hero 3 ™, GoPro, San Mateo, California, USA.

^8^Excel 2016, Microsoft, Redmond, WA.
