# Supplementary material for: Effect of blindfolding the lead resuscitator on frequency of closed-loop communication during veterinary cardio-pulmonary resuscitation training: a randomized, controlled pilot study
Source: Front Vet Sci. 2025 Jan 8;11:1484506. doi: 10.3389/fvets.2024.1484506 (PMC11750865; doi:10.3389/fvets.2024.1484506)
Supplement: Supplementary file 1 [file Data_Sheet_1.ZIP › Supplementary files .docx]

Supplementary material 1- Pre- and Post-study questionnaires

Pre scenario questionnaire questions

1. Are you aware of what closed-loop communication means?
2. In one sentence define what you think closed-loop communication means?
3. How often do you feel closed-loop communication is used in this practice; never, rarely, infrequently, often, always.
4. When did you last complete any CPR training? Please enter date if known
5. Role
6. Which department(s) do you spend the majority of your time in?
7. Name and surname
8. If you are a vet, how many years post-graduation are you? If you are not a vet, please type N/A
9. I confirm I have watched the CPR training video

Post scenario questionnaire

1. Are you aware of what closed-loop communication means?
2. In one sentence define what you think closed-loop communication means?
3. Did you feel this session was a helpful tool to learn about closed-loop communication?
4. How likely on a scale of 1-10 are you to use closed-loop communication in the future?
5. Please enter your name and surname
6. If you have any comments or feedback, please write in the comment box below

Supplementary material 2-Scenarios

**Researcher script to participants read by researcher and available as a written document to participants.**

**A consent form must be signed and returned to the researcher before filming begins.**

Thank you for taking part in these CPR simulations. You will be recorded, and your data will be kept securely and reviewed by the principal researcher. If you do not feel comfortable or wish to stop participating in the project at any point, please make the researchers aware.

Hospital health and safety protocols and current COVID protocols must be adhered to. Personal protective equipment (PPE) is available and must be worn during the simulations.

The simulation will be carried out for between 2-6 minutes and the researcher will let you know when the time has finished. After each simulation you will not be required to carry out a debrief within the group.

**Scenario 1 and 3**

A member of the public has rushed into the practice carrying this dog. They did not see what had happened to this dog but found it lying at the side of the road. It is not breathing and there is no pulse. The CPR code is presumed resuscitate until the owner can be contacted. The dog weighs 20kg. No further history is available.

**Scenario 2 and 4**

This dog has just returned to wards after radiographs of the limbs. It was found to be unresponsive, with no pulse and not breathing. The sedation sheet shows this 30kg dog received 50 micrograms of dexmedetomidine intramuscularly 20minutes ago and is a confirmed CPR code of closed chest resuscitation. There is no intravenous access or monitoring attached to the dog. The clinician is speaking with the owners.
